# Neural network analysis in pharmacogenetics of mood disorders

**DOI:** 10.1186/1471-2350-5-27

**Published:** 2004-12-09

**Authors:** Alessandro Serretti, Enrico Smeraldi

**Affiliations:** 1 Istituto Scientifico Universitario Ospedale San Raffaele, Department of Neuropsychiatric Sciences, Milano, Italy; 2 Università Vita-Salute San Raffaele, School of Medicine, Milano, Italy

## Abstract

**Background:**

The increasing number of available genotypes for genetic studies in humans requires more advanced techniques of analysis. We previously reported significant univariate associations between gene polymorphisms and antidepressant response in mood disorders. However the combined analysis of multiple gene polymorphisms and clinical variables requires the use of non linear methods.

**Methods:**

In the present study we tested a neural network strategy for a combined analysis of two gene polymorphisms. A Multi Layer Perceptron model showed the best performance and was therefore selected over the other networks. One hundred and twenty one depressed inpatients treated with fluvoxamine in the context of previously reported pharmacogenetic studies were included. The polymorphism in the transcriptional control region upstream of the 5HTT coding sequence (SERTPR) and in the Tryptophan Hydroxylase (TPH) gene were analysed simultaneously.

**Results:**

A multi layer perceptron network composed by 1 hidden layer with 7 nodes was chosen. 77.5 % of responders and 51.2% of non responders were correctly classified (ROC area = 0.731 – empirical p value = 0.0082). Finally, we performed a comparison with traditional techniques. A discriminant function analysis correctly classified 34.1 % of responders and 68.1 % of non responders (F = 8.16 p = 0.0005).

**Conclusions:**

Overall, our findings suggest that neural networks may be a valid technique for the analysis of gene polymorphisms in pharmacogenetic studies. The complex interactions modelled through NN may be eventually applied at the clinical level for the individualized therapy.

## Background

The increasing number of available genotypes for genetic studies in humans requires more advanced techniques of analysis [1]. Moreover, genes interact in a complex way, with some gene variants acting additively with others, in a multiplicative way or with a compensatory effect [2,3]. Traditional statistical techniques are not appropriate for detecting such effects [4], because they rely on the basic assumption of linear combinations only [5]. Investigation in multifactorial disorders in fact evidenced that non linear interactions are not detected by traditional regression analyses [6].

In particular, psychiatric disorders are characterized by a non mendelian, multifactorial genetic contribution with a number of susceptibility genes interacting with each other [7,8]. In the process of disentangling the contribution of environment versus genes, it has been recently suggested to focus on endophenotypes instead of psychiatric syndromes as a whole [9,10]. One interesting endophenotype is drug response, a field that gained much attention due to the possible clinical applications, ranging from individualized therapy to new drug development [11-14]. However, notwithstanding the promising results observed in the pharmacogenetic field, no single major effect gene was identified, but a variable number of polymorphisms in various genes are supposedly involved in modulating the response and/or side effects to drugs [15-20].

Since our initial study [21] we investigated the short term response to Selective Serotonin Reuptake Inhibitors (SSRIs) and a number of candidate genes, observing both positive and negative associations [22].

However, both the increasing number of genes associated with response and the limitations of traditional methods of analysis are factors requiring the use of new techniques of analysis that more closely resemble to the underlying biological process, i.e. that allows for non-linear interactions.

Neural networks (NN) have been proposed for such studies [1,23,24]. The main advantage of neural networks is that complex non-linear relationships can be modelled, potentially incorporating high-order interactions between predictive variables. This is of particular importance in a complex phenotype such as antidepressant response [22,25].

NN have been used in other fields of medicine, for example to predict cyclosporine dosage in patients after kidney transplantation [26], perspective outcome in AIDS research [27] but also in a genetic analysis in heart disease analysing 10 candidate genes simultaneously [28]. More complex models including gene-environment interactions have been developed [29].

In fact, neural networks proved to outperform single marker association tests, particularly in the case of a complex mode of inheritance or where multiple mutations result in more than one haplotype associated with the disease [25,30,31].

In the present paper we have re-analysed our sample where polymorphism in the transcriptional control region upstream of the 5HTT coding sequence (SERTPR) and in the Tryptophan Hydroxylase (TPH) gene were analized [32], in that paper we observed an association of both polymorphisms with drug response but we could not evaluate their possible non linear interactions. In the present paper we had the aim of evaluating the validity of NN models and of comparing them with traditional statistical techniques (multiple regression and discriminant function analysis).

## Methods

### Sample

The sample was already described in the original paper [32]. Briefly, two hundred and seventeen depressed inpatients were included in this study (age = 52.11 ± 12.04; onset = 37.97 ± 12.16; female/male: 144/73; bipolars: delusional/non delusional = 40/33, major depressives: delusional/non delusional = 71/73). All patients were evaluated at baseline and weekly thereafter until the sixth week using the 21-item Hamilton Rating Scale for Depression (HAM-D-21) [33] administered by trained senior psychiatrists blind to genetic data and to treatment (fluvoxamine 300 mg daily from day 8 plus pindolol 7.5 mg to one third of the sample). A decrease in HAM-D scores to 8 or less was considered the response criterion. After the procedure had been fully explained to all subjects, informed consent was obtained.

Plasma fluvoxamine levels were determined by high-performance liquid chromatography after 2 weeks of stable 300 mg daily dose [34]. Nine patients with extreme plasma levels (more than 2 standard deviations) were removed from the study in order to avoid biases due to side effects that are present at high doses, also subjects with plasma levels below 20 ng/ml were excluded as this may indicate non compliance, but no cases with such low doses were observed. The influence of both SERTPR and TPH polymorphisms was limited to subjects not taking pindolol [32] therefore we included in the present study the 121 subjects including fluvoxamine alone (81 responders/40 non responders). DNA analysis was performed as described in the original paper [32].

### Review of the models used

#### Multilayer Perceptrons

This is one of the most popular network architecture in use today, though relatively recent [35]. In MLP the units each perform a biased weighted sum of their inputs and pass this activation level through a transfer function to produce their output, and the units are arranged in a layered feedforward topology. The first step of the analysis is the choice of the number of layers and nodes. This is performed searching for a minimum in the error/performance hyperplane. Once the number of layers, and number of units in each layer, have been selected, the weight and threshold of the network must be set so as to minimize the prediction error made by the network. This is the role of the training algorithms. The best-known example of a neural network training algorithm is back propagation. In back propagation, the gradient vector of the error surface is calculated and used to decrease the error. A sequence of such moves (slowing as we near the bottom – epochs) will eventually find a minimum. A large number of epochs with no further improvement in the performance suggests that the optimum set of weights has been reached.

#### Linear Networks

Originally developed about 60 years ago by Fisher [36], in classification, the hyperplane is positioned to divide the two classes (a linear discriminant function) while in regression, it is positioned to pass through the data. A linear model is typically represented using an NxN matrix and an Nx1 bias vector. The linear network provides a good benchmark against which to compare the performance of your neural networks.

#### Radial Basis Function Networks

In a radial basis function network the response surface of a single radial unit is a Gaussian (bell-shaped) function, peaked at the center, and descending outwards. RBF networks have advantages and disadvantages over MLPs. First, they can model any non-linear function using a single hidden layer, which removes some design-decisions about numbers of layers. Second, the simple linear transformation in the output layer can be optimized fully using traditional linear modelling techniques, which are fast and do not suffer from problems such as local minima which plague MLP training techniques. However the clumpy approach also implies that RBFs are not inclined to extrapolate beyond known data: the response drops off rapidly towards zero if data points far from the training data are used, therefore they are less reliable for clinical samples such our one. Detailed review of the models are reported elsewhere [24,37].

### Model development and selection

An "intent-to-treat" analysis was carried out for all patients who had a baseline assessment and at least 1 assessment after randomization, with the last observation carried forward on the HAM-D. For the current application the inputs to the first layer of the neural network consist of SERTPR and TPH genotypes while the target outputs consist of response status. The network is then trained to attempt to predict response from genotypes. Each node of the input layer of the network is set to a value representing the genotype of each polymorphism. For each polymorphism and for each subject this value is set to genotypes aa, ab or bb. If a marker genotype is missing then the input is assigned a value equal to the average of the values for all subjects in the dataset, however no missing data were present in our sample. The target output for the network is set to 1 or 2 depending on whether the subject is responding or not.

The best network was selected on the basis of its discriminating error and performance, positive and negative predictive values were also reported for each model. This last was expressed as area under the Receiving Operator Characteristic (ROC) Curve. The area under a ROC curve ranges from zero to one, with values close to unity indicating better predictive power; an area of 0.5 indicates that the model is not predicting better than a random choice.

However, one major problem of NN analyses is to establish if the prediction from genotypes is greater than would be expected by chance. If the whole sample is used for training, the network will to some extent "learn to recognise" particular features of each member of the dataset and can use these to predict response in a way which may not reflect any general association between marker genotypes and disease. Generally, this problem is faced by a set of strategies: dividing the dataset (50:50, 80:20...), Jackknife, bootstrapping, cross-validation and so on. However those methods present some disadvantages, in particular if only a part of the data is used to train the network this leads to a loss of power given that subjects in the validating part have different patterns of association between genotypes and drug response.

In order to remedy these problems, in the case of MLP, it has been suggested to perform both training and testing on the entire dataset. The statistical significance of any observed association between outputs and affection status can be estimated using a permutation test [25].

Once the network was defined, a statistic, denoted T, is calculated to compare the outputs for responders and non responders in the same way as an unpaired t statistic, although the statistic is not expected to follow a t distribution under the null hypothesis. Instead, in order to estimate statistical significance a permutation procedure is performed. A large number of replicate data sets are generated from the original data and the obtained network model by randomly permuting genotypes with respect to affection status. For each of these replicate data sets we can then train and test the data set as before, each time calculating T. Since each permuted data set will have only random association between genotype and affection status we obtain N values of T which provide a distribution of T under the null hypothesis. We count the number of times any of these values exceeds the value of T we obtained for the real dataset and denote this number R. Then (R + 1)/(N + 1) provides an unbiased estimate of the statistical significance of the association between genotype and affection status in the real dataset.

In order to estimate a p-value of alpha, one should carry out approximately 10/alpha replicates. Typically, in order to detect association at a significance of 0.01 one would perform 1000 replicates (including the real dataset and 999 permuted datasets). In the case of the present paper we performed 10000 replicates.

Multiple regression and discriminant function analyses were performed to compare the results obtained with the NN strategy with traditional techniques. Responder status was the dependent variable with SERTPR and TPH as independent variables. Genotypes were scored in the following way according to the hypothesis of codominance (SERPR*l/l = 1, SERPR*l/s = 2, SERPR*s/s = 2, TPH*C/C = 1, TPH*C/A = 2, TPH*A/A = 2).

Calculations for the NN selection were performed using STATSOFT (Kernel release 5.5 A). Evaluation was performed using the NNPERM package [31].

## Results

**Figure 1:**
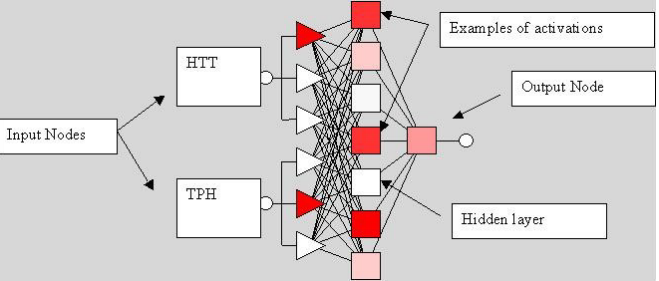
MLP composed by 1 hidden layer with 7 nodes used for the analysis.

MLP showed the best performance and was therefore selected over the other networks (see table 1). The MLP selected over the other models on the basis of error and performance was composed by 1 hidden layer with 7 nodes (Figure 1) after testing about 150 different MLP models. The network showed a very good basic performance (Error 0.430, Performance 0.685).

**Table 1:** Comparison of NN models. PPV = Predictive Positive Value, NPV = Predictive Negative Value, ROC = area under the ROC curve.

Network type	Error	Performance	sensitivity	specificity	PPV	NPV	ROC	Youden's J
Linear	0.447	0.636	67.12	56.34	75.97	45.46	0.687	0.23
RBF	0.449	0.691	85.61	35.21	73.10	54.35	0.664	0.21
MLP (1 – 7)	0.439	0.682	77.50	51.20	76.35	52.17	0.731	0.28

After, we trained the network with the back propagation algorithm. Initially we used a learning rate of 0.1 (momentum 0.3, noise set to 0), after 5000 epochs we reduced it to 0.01 but after 5000 further epochs we observed no improvement and therefore we finished the selection process and retained the network. Both polymorphisms contributed substantially to the model (SERTPR error= 0.532, ratio = 1.21; TPH error= 0.450, ratio = 1.02). This was expected since both markers were individually associated with response. In detail single marker significance, calculated as simple allelic chi-square, was p = 0.00058 for SERTPR and p = 0.025 for TPH. The classification of subjects in responders and non responders was 77.5 % for responders and 51.2% for non responders. Classification may vary depending from the selected threshold, therefore the area under the ROC curve is a better indicator of performance, in this case the area was 0.731. We also evaluated the predictive power of the network with the SERTPR polymorphism only, in this case the area under the ROC curve was 0.698. We may therefore observe that the add of TPH polymorphism increases the predictive power of the system.

In order to evaluate the significance of the network we applied a permutation test with 10000 replicates. The t statistic for the network was 4.35, it was achieved in 81 out of 10000 simulations yielding a network p-value = (81+1)/(10000+1) = 0.0082.

Finally, we performed a comparison with traditional techniques. A multiple regression analysis showed a significant correlation (p = 0.0004) with a variance explained of 12.5%. The discriminant function analysis correctly classified 34.1 % of responders and 68.1 % of non responders (F = 8.16 p = 0.0005).

Following, we tested the possible impact of clinical variables on response. We included in the model the following variables: Age, age at onset, sex, education, diagnosis, presence of delusional features, recurrence index (defined as number of episodes per year), pindolol augmentation and baseline HAM-D. With those variables no satisfactory network was identified. They were therefore not considered as possible confounding factors in the genetic analysis.

## Discussion

This paper reports the first attempt to use NN in pharmacogenetic analyses. We applied this technique to short term antidepressant response in mood disorders. Our analyses suggest that MLP network is the most appropriate for this kind of data, in according with previous observations [25]. The growing number of polymorphisms (about 3.000.000) and the growth of simultaneous techniques such as gene arrays ask for appropriate techniques of analysis. Traditional ones have strong limitations not allowing for non linear interactions and the risk of overfitting in the case of multiple polymorphisms analysed in necessary limited sample sizes. We observed that a relatively simple MLP NN is able to predict response in a way comparable to traditional techniques. The lack of non linear interactions in the simple model we analysed [32] explains why did not observe a marked superiority of NN over traditional analyses. However the most promising result of the strategy we tested in the present paper is the possibility to add a large number of polymorphism to the network and to evaluate the improvement in the prediction, showed by the area under the ROC curve. Moreover the significance of the network can be evaluated with the permutation test [25,31]. Moreover the MLP model we used is quite parsimonious in terms of parameters used (2 input variables, 1 output variable and 1 hidden layer with 7 nodes).

Further developments of this strategy are the inclusion of more detailed information on the phenotypic side. The classification results we obtained are not sufficient in clinical terms were in particular much higher specificities are needed in order to recognize in advance non responders. To reach this target we should consider that we previously observed that some polymorphism influence only part of the whole depressive symptomatology [38]. Further clinical variables should also be considered as reported to influence the short term antidepressant outcome [39], even if previous NN studies failed to identify clinical predictors of antidepressant response [40]. Our analyses are in line with this view, in fact the clinical variables we analysed were not significantly associated with outcome.

The relatively small sample we used does not guarantee against a possible overfitting phenomenon, therefore enlargement of the sample is a priority. Moreover we used the same sample for testing and validating our result, this is not a standard technique [41], this problem is usually faced by a set of strategies such as dividing the dataset, Jackknife, bootstrapping, cross-validation and so on. However those methods present some disadvantages, in particular if only a part of the data is used to train the network this leads to a loss of power in the case that subjects in the validating part have different patterns of association between genotypes and drug response. Therefore in the present paper we performed both training and testing on the entire dataset with the use of a permutation test to validate the results [25]. Another limitation of the present paper is that we compared NN with multiple regression only, other techniques could be tested as well such as set association [30], multifactor dimensionality reduction [42], and logic regression [43].

Differences in allele frequency for different populations have been reported [44]. However our sample was composed of subjects mainly collected in the North of Italy with Italian antecedents for at least two generations, though genetic heterogeneity have been evidenced for some isolate populations (such as Sardinia, not included in our sample) Italy is characterized by a substantial genetic homogeneity [45]. Another caveat is linked to the characteristics of our sample. In fact the Center for Mood Disorders of San Raffaele Hospital is a tertiary structure, therefore we cannot exclude a potential selection bias associated with illness severity and possible extension to outpatients or drug abusers are not warranted [46].

## Conclusions

Overall, our findings suggest that neural networks may be a valid technique for the analysis of gene polymorphisms in pharmacogenetic studies. The complex interactions modelled through NN may be eventually applied at the clinical level for the individualized therapy [47].

## Competing interests

The author(s) declare that they have no competing interests.

## Authors' contributions

AS conceived the study, drafted the manuscript and participated in the design of the study and performed the statistical analysis. ES participated in its design and coordination. All authors read and approved the final manuscript

## Pre-publication history

The pre-publication history for this paper can be accessed here:


